# Factors Associated with Family Planning Status and Voluntary Childlessness in Women of Childbearing Age with Inflammatory Bowel Diseases

**DOI:** 10.3390/jcm12134267

**Published:** 2023-06-26

**Authors:** Christian P. Selinger, Helen Steed, Satvinder Purewal, Rebecca Homer, NIHR BioResource, Matthew Brookes

**Affiliations:** 1 Leeds Gastroenterology Institute, Leeds Teaching Hospitals, Leeds LS9 7TF, UK; 2 Leeds Institute of Medical Research at St James’s, University of Leeds, Leeds LS9 7TF, UK; 3 Department of Gastroenterology, Royal Wolverhampton Hospital, Wolverhampton WV10 0QP, UK; helen.steed@nhs.net (H.S.); matthew.brookes@nhs.net (M.B.); 4 Gastroenterology, University of Wolverhampton, Wolverhampton WV1 1SG, UK; satvinder.purewal@wlv.ac.uk (S.P.); r.homer3@wlv.ac.uk (R.H.); 5 NIHR BioResource, Cambridge University Hospitals, Cambridge Biomedical Campus, Cambridge CB2 0QQ, UK

**Keywords:** inflammatory bowel disease, pregnancy, family planning, voluntary childlessness

## Abstract

Background: Women with Inflammatory Bowel Diseases (IBD) have fewer children and stay childless more often. The decision-making process around family planning choices remains incompletely understood. Methods: We examined family status in women who at recruitment to the UK IBD Bioresource had not had children yet via an electronic survey. The primary outcome was the proportion of women with voluntary childlessness. Secondary outcomes were factors associated with family planning status. Results: Of 326 responders, 10.7% had either given birth, were currently pregnant or were currently trying to conceive; 12.6% were planning to conceive within 12 months; 54.4% were contemplating conception in the distant future (vague plans); and 22.3% were voluntarily childless. Factors associated with family planning status fell into three areas: general background (age, household income, perceived support to raise a child), relationship status (sexual orientation, being single, not cohabiting, perception of being ‘in the right relationship to raise a child’, perception of a good sex life) and the expression of having a child as a goal in life. On binary logistics regression analysis with voluntary childlessness versus vague family plans as the outcomes of choice, having a household income of <£30,000 (*p* = 0.046), not seeing a child as a life goal (*p* < 0.0001) and identifying as lesbian or bisexual (*p* = 0.047) were independent predictors of voluntary childlessness. Conclusions: Clinicians should consider sexual orientation, income, younger age, current relationship and lack of expression of having a child as a life goal as important factors for family planning when providing care. Pre-pregnancy advice should be made widely available for women with IBD.

## 1. Background

Inflammatory Bowel Diseases (IBD) are chronic inflammatory conditions affecting the gastrointestinal tract [1]. Patients experience symptoms including diarrhoea, rectal bleeding, abdominal pain, fatigue, fistula discharge and extra-intestinal symptoms affecting the skin, eyes and joints [1]. While ulcerative colitis is a mucosal disease limited to the rectum and colon, Crohn’s disease affects the gastrointestinal tract anywhere between the mouth and anus. While medical treatments have become more effective over the last two decades, surgery is still frequently required [1]. Frequently, patients with IBD are diagnosed in early adolescence or as young adults. IBD is therefore present in many women of childbearing age [2]. Women with IBD have fewer children than healthy controls [3], and voluntary childlessness (deciding not to have children) is significantly more common in patients with IBD [4,5]. Previous studies have shown that many women of childbearing age harbour concerns about potential medication side effects on the unborn [6], inheritance of IBD by the infant and effects of IBD on pregnancy and vice versa [6,7,8]. Many women of childbearing age have poor knowledge of IBD- and pregnancy-related issues [9,10,11], which is associated with views contrary to evidence-based medical guidelines [7].

Our previous work has suggested that poor patient knowledge, older age, unemployment, being single and not seeking medical advice about IBD and pregnancy were associated with voluntary childlessness [5]. The decision-making process around family planning is complex and patients undergo a similar process as when coming to terms with getting diagnosed with IBD [12]. Patients may weigh the effects of IBD on their ability to look after children, perceived difficulties of coping with a pregnancy, concerns over IBD medication, inheritance concerns and financial and personal circumstances when deciding on whether to start a family [8]. The decision-making process around this remains insufficiently understood. Previous studies have mainly focussed on women who had already been pregnant or focussed on analysing those with voluntary childlessness compared with those having children. There have so far not been any studies examining longitudinal trends in the same patient cohort. 

We therefore examined family planning status in a group of women from the UK IBD BioResource cohort, who did not have children at inclusion. For this analysis, we focussed on the baseline factor associated with family planning status while longitudinal follow-up of the cohort was ongoing.

## 2. Methods

### 2.1. Study Cohort

Recruitment to the UK IBD BioResource—an open research platform—started in 2016 [13]. So far, over 35,000 patients from over 100 UK hospitals have been included during routine hospital visits for their IBD care. At recruitment, patients provide baseline data on their social and medical background. Disease and treatment characteristics are collected from medical records at baseline and blood is sampled for genetic analysis [13]. Participants are frequently contacted for further studies nested within the UK IBD BioResource cohort [14]. For this study, we approached 2399 female patients aged 18–45 years, who at recruitment to UK IBD BioResource between 2016 and 2021 had not had children. 

### 2.2. Recruitment and Study Procedures

Potential participants were contacted by email on up to three occasions between March 2022 and July 2022. Responders were invited to fill in an electronic survey hosted on the secure REDCap (Research Electronic Data Capture) research environment. Study data were collected and managed using REDCap electronic data capture tools hosted at Leeds Teaching Hospitals [15,16]. REDCap is a secure, Web-based software platform designed to support data capture for research studies. Participants provided informed consent after reading the online patient information leaflet by continuing to the research questionnaire and answered questions around current relationship status, current reproduction, future reproduction intentions, current disease state and current IBD medication. Family planning status was categorised as currently pregnant or currently trying to conceive, aiming to conceive within the next year, vague plans to have children in more distant future and identifying as voluntarily childless. Medication adherence was assessed using a visual analogue scale (VAS; 0–100) which was validated for IBD, with a cut-off of ≥80 deemed as adherent [17]. We assessed current psychological state with the Hospital Anxiety and Depression Scale (HADS) [18]. Pregnancy-related IBD knowledge was assessed by the Crohn’s and Colitis Pregnancy Knowledge Score (CCPKnow), a validated self-assessment tool with scores ranging from 0 to 17 and scores ≥ 8 deemed adequate [9]. Demographic and phenotype type data were extracted from the UK IBD BioResource.

### 2.3. Outcomes

The main outcome of this baseline analysis of the cohort was the proportion of women with voluntary childlessness. Secondary outcomes were factors associated with family planning status, including a binary regression analysis of factors with voluntary childlessness compared with those with vague family plans. 

### 2.4. Statistical Analysis Plan

Descriptive statistics were reported as mean or proportions. Categorical data were compared between groups using Chi-square test, whereas independently sampled t-test was used for normally distributed continuous variables and Mann–Whitney U test for non-normally distributed continuous variables. We performed a binary regression analysis to determine factors independently associated with medication adherence. Differences were considered statistically significant if *p* < 0.05. IBM SPSS version 25 was used for statistical analysis.

### 2.5. Ethical Considerations

The UK IBD BioResource was reviewed and approved by Cambridge Central Research Ethics Committee (ref 15/EE/0286). All patients provided written informed consent at initial recruitment. For this nested phase 2 study within the UK IBD BioResource, HREC approval was granted by London–Stanmore Research Ethics Committee (ref 19/LO/1891). Participants in this study provided consent by progressing from the patient information leaflet to the questionnaire. 

## 3. Results

### 3.1. Cohort Composition, Disease and Treatment Characteristics

**Figure 1:**
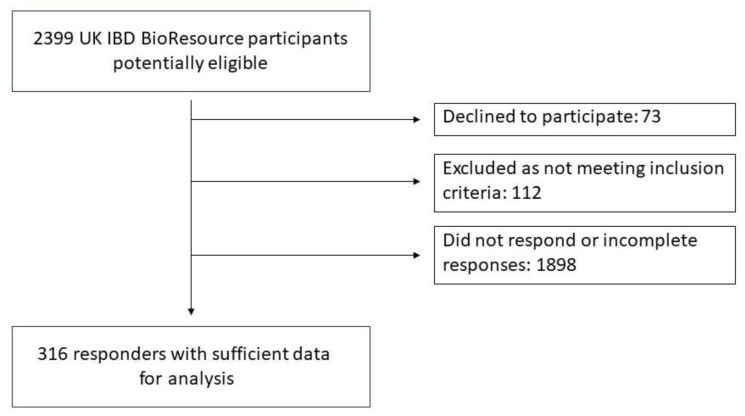
Participant flow chart.

We invited a total of 2399 UK IBD BioResource participants, 73 of whom declined and 112 were found to be ineligible. We received 316 (13.2%) responses with sufficient data for analysis (Figure 1). Respondents were aged 18–34 years and predominantly of white British ethnicity (Table 1). Disease phenotype, surgical history and current treatment are displayed in Table 2. There were 33 (10.7%) women who had either already given birth, were currently pregnant or were currently trying to conceive, and 39 women (12.6%) were planning to conceive within 12 months. The remainder consisted of 168 (54.4%) who were contemplating conception in the distant future (vague plans; >3 years) and 69 (22.3%) who were not planning to have children (voluntary childlessness). 

Percentages were calculated for the whole cohort for diagnosis and medical treatment. For phenotype, surgery and stoma data the percentages were calculated for the CD and the UC/IBD—U cohort separately. Where data do not add for the total cohort, we omitted missing data.

### 3.2. Disease, Treatment and Demographic Factors Associated with Family Planning Status

Ethnicity, IBD type, IBD phenotypes, presence of perianal disease, self-reported IBD disease activity, hospital admission within 12 months, exposure to different types of IBD medication, presence of extra-intestinal manifestations, previous IBD surgery, anxiety and depression were not associated with family planning status. 

Although the data are complex, overall, our findings suggest that those who have vague and/or no plans for children are more likely to be younger, less economically advantaged, in full-time education, single or lesbian/bisexual. Patients with vague family plans were more often below < 30 years of age (85.1%) compared with those pregnant or aiming to conceive (75%), those planning to conceive in 12 months (67.7%) and those with voluntary childlessness (76.7%; *p* = 0.004). Those who had vague family plans (36.8%) and those with voluntary childlessness (52.6%) had a household income of <£30,000 more often than those pregnant or trying (29.6%) and those planning pregnancy for the next year (38.8%; *p* = 0.0008). Women who were pregnant/currently trying to conceive were less likely to have university-level education (*p* = 0.023), but there were no differences between the three other family status groups. Employment levels were high amongst all four family planning status groups, but women with vague family planning plans (13.7%) and those with voluntary childlessness (11.8%) were more likely to be in full-time education (others 1.4%; *p* < 0.001). Patients with voluntary childlessness were more likely to be single (43.3%) and less likely to be cohabiting (34.4%) compared with those with vague family plans (single 34.55, cohabiting 41.1%), those planning to conceive in the next 12 months (single 0%, cohabiting 94.9%) and those currently pregnant or aiming to conceive (single 6%, cohabiting 82%; *p* < 0.001). Participants with voluntary childlessness identified as lesbian or bisexual more often (29.5%) than those currently pregnant/trying for pregnancy (10%), those planning for pregnancy within 12 months (7.9%) and those with vague family plans (12.6%; *p* = 0.0025).

Data suggest that patients who are pregnant or aiming to conceive are, on the whole, more likely to demonstrate behavioural and knowledge patterns consistent of family planning preparations. For example, patients currently pregnant or aiming to conceive (67%) and those planning for pregnancy within 12 months (51.3%) were significantly more likely to have spoken to a health care professional about IBD and pregnancy than those with vague plans (20.2%) and those with voluntary childlessness (13%; *p* < 0.001; difference between vague plans and voluntary childlessness was not significant). Patients planning for a pregnancy in the next year were significantly more likely to be adherent (59.0%) compared with those who had actively aimed for a pregnancy (previous pregnancy, currently pregnant and currently aiming to conceive; 33.3%), those planning for children in the more distant future (38.7%) and those not planning to have children (29.0%; *p* = 0.019). Patients planning to conceive had significantly higher CCPKnow scores than those meeting the criteria for the other family planning categories (median eight vs. seven; *p* = 0.035). There were no significant differences in adherence and CCPKnow between those with vague plans and those with voluntary childlessness.

### 3.3. Patient View Factors Associated with Family Planning Status

Patients’ view on the ability of their body to cope with a pregnancy, their ability to look after a child and concerns about potential inheritance of IBD were not associated with family planning status.

Patients who were currently pregnant or aiming to conceive were more likely to report life events or lifestyle factors associated with perceived ‘stable’ conditions for parenting. For example, patients currently pregnant or aiming to conceive (84.8% and those planning for pregnancy within 12 months (82%) more often felt that they had sufficient financial funds to raise a child than those with vague plans (50.6%) and those with voluntary childlessness (34.8%; *p* < 0.001 for overall comparison; difference between vague plans and voluntary childlessness *p* = 0.027). Patients currently pregnant or aiming to conceive (97%) and those planning for pregnancy within 12 months (97.5%) more often felt that they were in the ‘right relationship’ to raise a child than those with vague plans (35.2%) and those with voluntary childlessness (17.3%; *p* < 0.001 for overall comparison; difference between vague plans and voluntary childlessness *p* = 0.007). Patients currently pregnant or aiming to conceive (84.8%) and those planning for pregnancy within 12 months (92.3%) more often expressed that raising a child was a life goal for them than those with vague plans (71.4%) and those with voluntary childlessness (7.2%; *p* < 0.001 for overall comparison; difference between vague plans and voluntary childlessness *p* < 0.001). Patients currently pregnant or aiming to conceive (81.8%) and those planning for pregnancy within 12 months (82%) rated their sex life as good more than those with vague plans (63.7%) and those with voluntary childlessness (60.1%; *p* = 0.045). 

Participants with voluntary childlessness less often felt that they had sufficient support to raise a child (68.1%) than those currently pregnant/trying for pregnancy (93.9%), those planning for pregnancy within 12 months (84.6%) and those with vague family plans (80.9%; *p* = 0.014; difference between vague plans and voluntary childlessness *p* = 0.03).

### 3.4. Binary Regression Analysis, Voluntary Childlessness versus Vague Plans

On binary logistics regression analysis with voluntary childlessness versus vague family plans as the outcomes of choice, having a household income of <£30,000 (*p* = 0.046; Table 3), not seeing a child as a life goal (*p* < 0.0001) and identifying as lesbian or bisexual (*p* = 0.047) were independent predictors of voluntary childlessness. 

## 4. Discussion

We examined family planning status in an enriched group of women, who at the time of recruitment to the UK IBD Bioresource had not had children yet. This allowed us to study factors associated with family planning decisions. In contrast to other studies, the percentage of women with voluntary childlessness appears higher, but is likely explained by the fact that we did not aim to include those that had already given birth. 

Interestingly, many disease- and treatment-related factors were not associated with family planning status. It appears that disease severity, different disease phenotypes and exposure to different medications did not have a deciding influence on women’s plans regarding potential pregnancy and having children. We previously assumed based on indirect evidence [5] that disease severity may impact family planning. However, our data could reassure IBD clinicians that direct IBD effects are not likely a key driver of voluntary childlessness. Women have expressed concerns about the effect of pregnancy on IBD and vice versa, as well as the inheritance of IBD, but our data suggest that while these concerns affect women with IBD, they do not seem to drive decision making [6,8,19]. Furthermore, a shortcoming of the existing literature is the predominant focus on disease or biological factors in determining family planning decisions and insufficient emphasis on considering non-medical aspects of the participants’ lives. Participants’ psychosocial and subjective life experiences were also key in determining their family planning decisions. 

Specifically, we found that disease- and treatment-related factors were not associated with family planning; however, some demographics and life style/personal factors were associated. The factors that were associated with family planning status covered three broad areas. The first area concerned the general background. Our findings suggest that patients who had a vague plan and/or were choosing voluntary childlessness were more likely to be younger, less economically advantaged (earning £30<), in full-time education, single or lesbian/bisexual compared with other groups. The second group, those who were pregnant or aiming for children, were more likely to perceive their personal situation as favourable for parenting. For example, they were more likely to perceive that they had sufficient financial funds to raise a child, more likely to believe they were in the ‘right relationship’ to raise a child, more likely to express that raising a child was a life goal for them and also rated their sex life as good more often than those with vague plans and those with voluntary childlessness. The third area that directly related to family planning status was the expression of having a child as a goal in life.

Furthermore, those wanting to have a child demonstrated more behavioural and knowledge patterns associated with parenting. For example, they had higher levels of interaction with health care professionals about IBD and pregnancy, were more likely to be adherent to medication and had higher CCPKnow scores. Previous studies have shown that interactions with HCPs are associated with better disease-related patient knowledge [5,10]. Similarly, higher CCPKnow scores were associated with childbearing [20]. We postulate that women may only interact with health care professionals about IBD and pregnancy when having children becomes a more concrete plan for them. Our longitudinal follow-up of this cohort will provide further insight on this aspect. 

Further, HCPs need to consider that their patients may be making family planning decisions based upon their perceived ‘favourable’ life situations (e.g., ‘right’ partner, good finances), which may or may not coincide with the HCP’s medical advice on the timing of pregnancy (e.g., relapse). 

Sexual orientation was significantly associated with family planning status. Participants in our study identified more often as lesbian or bisexual (13.7%) than reported by the Office of National Statistics 2021 survey for the same groups (4.5%). Participants identifying as lesbian or bisexual were more often voluntarily childless than women identifying as heterosexual. Cohort studies from the US and Europe have shown that individuals identifying as lesbian or bisexual had significantly lower parenting desire and parenting likelihood [21,22].

When narrowing the analysis down to factors that differentiate women with voluntary childlessness from those with vague future family plans, we found that low household income, not seeing a child as a life goal and sexual orientation were independent predictors of voluntary childlessness. 

The results of our study are strengthened by the examination of a well-defined multi-centre cohort of women with IBD. We focused on patients for whom family planning decisions were most relevant. The multi-centre approach applied by the UK IBD Bioresource reduced factors often seen in single-centre studies. The limitations to our work include a survey design with a relatively low response rate, although low response rates are not uncommon amongst IBD survey studies on reproductive health [8]. As family planning status may be fluid, this baseline analysis may not show the full picture of how women with IBD make decisions on family planning. Longitudinal follow-up will aim to close the current gaps in our understanding.

Our findings do not provide a definitive understanding of the factors influencing family planning for women with IBD yet, but should nevertheless inform clinicians while longitudinal results are awaited. Clinicians should consider that family planning is not related to the course or severity of the women’s IBD. This highlights the importance of pre-pregnancy counselling to increase the chances of conceiving during times of permission, which is associated with better pregnancy outcomes [23]. Importantly, clinicians should not assume that women experiencing a more complicated course of IBD may not wish for a pregnancy. While many clinical consultations focus on the clinical aspects of IBD, asking considerate questions around relationship status and potential family plans could enable important discussions around fertility, potential pregnancy management and allow for timely pre-pregnancy counselling.

In conclusion, we found that sexual orientation, general background of income and age, the current relationship and the expression of having a child as a life goal were associated with family planning status and voluntary childlessness.

## Figures and Tables

**Table 1:** Patient demographics and social background.

Parameter	N=	Percentage
**Age**		
18–24 years	73	23.4%
25–29 years	172	55.1%
30–34 years	67	21.5%
**Ethnicity**		
White or White British	274	91.3%
Asian or British Asian	13	4.3%
Mixed	13	4.3%
**Sexual orientation**		
Straight/heterosexual	255	82.8%
Lesbian/homosexual	3	1.0%
Bisexual	39	12.7%
Other	11	3.6%
**Relationship status**	78 12 64 120 42	24.7% 3.8% 20.3% 38.0% 13.3%
Single, not currently dating		
Currently dating (<3 months)		
In relationship (>3 months) but not living together		
Living with partner unmarried		
Married		
**Family planning status**	33 39 168 69	10.7% 12.6% 54.4% 22.3%
Already given birth to a child or pregnant or currently aiming to conceive		
Planning to conceive within 12 months		
Contemplating conception in distant future		
Not planning to have children		
**Household income**		
<£10,000	13	5.0%
£10,000–£20,000	34	13.0%
£20,001–£30,000	52	19.9%
£30,001–£50,000	82	31.4%
>£50,000	80	30.7%
**Highest educational achievement**		
Secondary school	28	8.9%
Apprentice	92	29.1%
Bachelor’s	139	44.0%
Master’s/Doctorate	57	18.0%
**Employment**		
Working full time	227	71.8%
Working part time	39	12.3%
Full-time education	32	10.1%
Unemployed	15	4.7%
House person	3	0.9%

**Table 2:** Disease phenotype and treatment.

Parameter	N=	Percentage
**Diagnosis**		
Crohn’s Disease (CD)	175	55.4%
Ulcerative Colitis	123	38.9%
IBD—Unclassified	13	4.1%
Other	5	1.6%
**Crohn’s extent**		
Oesophageal-gastric	3	1.7%
Duodenal	5	2.9%
Jejunal	9	5.1%
Ileal	119	68.0%
Colonic	108	62.0%
Rectal	28	16.0%
**Crohn’s behaviour**		
Inflammatory	122	69.7%
Stricturing	26	14.9%
Penetrating	16	9.1%
**Crohn’s perianal disease**		
Ever present	49	28.0%
**Surgery**		
In patients with CD	49	28.0%
Stoma present	13	7.4%
**UC/IBD—U extent**		
E1 proctitis	18	13.2%
E2 left-sided disease	45	33.0%
E3 extensive colitis	48	35.3%
**UC/IBD—U surgery**		
Colectomy	13	9.6%
Pouch formed	6	4.4%
**Extra intestinal manifestations**	210 4 12 12 3 7 15 0	66.5%
None		
Primary sclerosing cholangitis		
Enteropathic arthropathy		
Erythema nodosum		
Iritis		
Orofacial granulomatosis		
Psoriasis		
Ankylosing Spondylitis		
**Current self-reported disease activity**		
Remission	96	30.8%
Mild	121	38.8%
Moderate	74	23.7%
Severe	21	6.7%
**IBD-related hospital admission in last 12 months**		
Yes	38	12.2%
**Current medical treatment**		
Mesalazine	90	28.5%
Thiopurines	94	29.7%
Methotrexate	10	3.2%
Anti-TNF	103	32.6%
Vedolizumab	38	12.0%
Ustekinumab	30	95.0%
Tofacitinib	5	1.6%
Steroid exposure in last 12 months	77	24.4%

**Table 3:** Binary regression analysis, voluntary childlessness versus vague plans.

	Vague Plans	Voluntary Childlessness	Categorical Analysis *p*-Value	Binary Regression B-Value	Binary Regression *p*-Value
Household income < £30,000	36.8%	52.6%	0.041	−0.893	0.046
“I have enough money to raise a child”	50.6%	34.8%	0.026	−0.521	0.267
“I’m in the right relationship to raise a child”	35.2%	17.4%	0.007	−0.015	0.977
“Having a child is a life goal for me”	71.4%	7.2%	<0.0001	−3.144	0.000
“I have enough support to raise a child”	80.1%	68.1%	0.032	−0.259	0.594
Lesbian or bisexual	11.2%	29.5%	0.001	1.083	0.047

## Data Availability

Summary data are available on reasonable request. Owing to ethics/consent for this study, primary data are not openly available.
